# The Bruininks-Oseretsky Test of Motor Proficiency-Short Form is reliable in children living in remote Australian Aboriginal communities

**DOI:** 10.1186/1471-2431-13-135

**Published:** 2013-09-06

**Authors:** Barbara R Lucas, Jane Latimer, Robyn Doney, Manuela L Ferreira, Roger Adams, Genevieve Hawkes, James P Fitzpatrick, Marmingee Hand, June Oscar, Maureen Carter, Elizabeth J Elliott

**Affiliations:** 1 Discipline of Paediatrics and Child Health, The University of Sydney, The Children's Hospital at Westmead, Clinical School, Locked Bag 4001, Westmead, NSW 2145, Australia; 2 The George Institute for Global Health, Sydney Medical School, University of Sydney, PO Box M201, Missenden Rd, Sydney 2050, Australia; 3 Poche Centre for Indigenous Health, Sydney School of Public Health, The University of Sydney, Sydney, NSW 2006, Australia; 4 Physiotherapy Department, Royal North Shore Hospital, Sydney, Australia; 5 School of Public Health, Curtin University of Technology, Perth, Australia; 6 Sydney Medical School, University of Sydney, Sydney, Australia; 7 School of Physiotherapy, University of Sydney, Sydney, Australia; 8 Western Australia Country Health Services, Derby, Australia; 9 University of Notre Dame, Broome, Australia; 10 Marninwarntikura Women’s Resource Centre, Fitzroy Crossing, Australia; 11 Nindilingarri Cultural Health Services, Fitzroy Crossing, Australia; 12 The Sydney Children’s Hospital Networks (Westmead), Westmead, Australia

**Keywords:** Fetal alcohol spectrum disorders, Fetal alcohol syndrome (FAS), Alcohol related neurodevelopmental disorder, Australian Aborigine, Maternal use of alcohol, School-aged children, Reproducibility of results, Culture, Motor skills, Child development

## Abstract

**Background:**

The Lililwan Project is the first population-based study to determine Fetal Alcohol Spectrum Disorders (FASD) prevalence in Australia and was conducted in the remote Fitzroy Valley in North Western Australia. The diagnostic process for FASD requires accurate assessment of gross and fine motor functioning using standardised cut-offs for impairment. The Bruininks-Oseretsky Test of Motor Proficiency, Second Edition (BOT-2) is a norm-referenced assessment of motor function used worldwide and in FASD clinics in North America. It is available in a Complete Form with 53 items or a Short Form with 14 items. Its reliability in measuring motor performance in children exposed to alcohol *in utero* or living in remote Australian Aboriginal communities is unknown.

**Methods:**

A prospective inter-rater and test-retest reliability study was conducted using the BOT-2 Short Form. A convenience sample of children (n = 30) aged 7 to 9 years participating in the Lililwan Project cohort (n = 108) study, completed the reliability study. Over 50% of mothers of Lililwan Project children drank alcohol during pregnancy. Two raters simultaneously scoring each child determined inter-rater reliability. Test-retest reliability was determined by assessing each child on a second occasion using predominantly the same rater. Reliability was analysed by calculating Intra-Class correlation Coefficients, ICC(2,1), Percentage Exact Agreement (PEA) and Percentage Close Agreement (PCA) and measures of Minimal Detectable Change (MDC) were calculated.

**Results:**

Thirty Aboriginal children (18 male, 12 female: mean age 8.8 years) were assessed at eight remote Fitzroy Valley communities. The inter-rater reliability for the BOT-2 Short Form score sheet outcomes ranged from 0.88 (95%CI, 0.77 – 0.94) to 0.92 (95%CI, 0.84 – 0.96) indicating *excellent* reliability. The test-retest reliability (median interval between tests being 45.5 days) for the BOT-2 Short Form score sheet outcomes ranged from 0.62 (95%CI, 0.34 – 0.80) to 0.73 (95%CI, 0.50 – 0.86) indicating *fair to good* reliability. The raw score MDC was 6.12.

**Conclusion:**

The BOT-2 Short Form has acceptable reliability for use in remote Australian Aboriginal communities and will be useful in determining motor deficits in children exposed to alcohol prenatally. This is the first known study evaluating the reliability of the BOT-2 Short Form, either in the context of assessment for FASD or in Aboriginal children.

## Background

### Introduction

In 2010, Aboriginal communities in remote north Western Australia initiated Australia’s first study of the prevalence of Fetal Alcohol Spectrum Disorders (FASD) to better understand the support services required to assist children and their families into the future [1]. This study, called the Lililwan Project, arose following concerns from Aboriginal leaders about the effect that high-risk drinking was having on the development of children within their communities [1] and the potential for FASD. FASD refers to a spectrum of lifelong physical, behavioural and neurodevelopmental disorders resulting from brain injury caused by prenatal alcohol exposure (PAE) [2,3]. Clinicians have suspected 30% or higher of the population in some remote Australian Aboriginal communities may have FASD where drinking rates are high [4]. The Lililwan Project will provide the first data for these communities.

### Diagnostic process

Diagnosis of FASD is complex, involving assessment for facial dysmorphology, growth deficiency and central nervous system (CNS) impairment or structural abnormalities. CNS impairment may manifest as deficits in memory, cognition, executive function, adaptive behaviour, sensory processing and language, as well as deficits in fine motor (FM) and gross motor (GM) function [5,6]. Current diagnostic systems for FASD include the University of Washington: The 4-digit Diagnostic Code [5], Canadian Guidelines [6], the Institute of Medicine [2] and the Centres for Disease Control and Prevention [7]. These systems agree on many aspects including the assessment of FM skills but only some include assessment of GM skills [5-7]. Physical activities are central to Australian Aboriginal culture hence inclusion of GM assessment within FASD diagnostic procedures captures a culturally relevant aspect of CNS function for children growing up in the Fitzroy Valley.

The Canadian Guidelines were applied to determine the prevalence of FASD amongst the children in the Lililwan Project cohort (n = 108). They require the assessment of both GM and FM functioning with standardised assessment tools using predefined cut-offs for impairment at 2 standard deviations (SD) below the population mean (< 3rd percentile) [6]. Within the diagnostic framework, these skills are assessed during the evaluation of nine domains of CNS impairment. GM and FM functioning fall into the first of these domains under the category of these domains under the category of *hard and soft neurologic signs (including sensory motor signs)*.

Recommendations exist within some international FASD diagnostic criteria [5,6,8] regarding appropriate standardised assessment tools to test motor proficiency in children with PAE but further guidelines are needed regarding age and cultural suitability. Other elements which need consideration in assessment tool selection are validity, established reliability in children with PAE, ability to assess mild to moderate motor impairment, and, as FASD is now recognised by the World Health Organisation as the leading preventable non-genetic cause of mental retardation [9], the tool must be able to be accurately administered in the presence of intellectual impairment. Furthermore, to satisfy FASD diagnostic cut-offs, assessment outcomes need to be reported in percentile ranks or standard deviations.

### Motor tool selection

To determine the most appropriate standardised assessment tool for measuring motor skills in the Lililwan Project cohort (i) a literature review was conducted; (ii) national paediatric physiotherapy networks were canvassed through a phone survey by contacting all of the Children’s Hospitals within Australia (n = 6); and (iii) representatives of national and international FASD networks were surveyed during informal discussions at the 4th International Conference on FASD, Vancouver, March 2011.

A comprehensive literature review for children aged 7 – 9 years of age revealed five studies in which GM performance was included in the motor assessment of children with a FASD diagnosis or with prenatal exposure to alcohol [10-14]. These studies used six different standardised GM assessment tools ie: Griffith Mental Developmental Scale (GMDS) [10], Pediatric Early Elementary Examination Second Edition (PEEX2) [11], Pediatric Examination of Educational Readiness Second Edition (PEERAMID 2) [11], Clinical Observations of Motor and Postural Skills (COMPS) [12], Movement Assessment Battery for Children (Movement ABC) [12], Modified Bruininks-Oseretsky Test of Motor Proficiency (BOTMP) [13] and McCarthy Scales of Children’s Abilities (MSCA) [14]. On further investigation only the Movement ABC and BOTMP were found to be comprehensive motor assessments. Recommendations from FASD diagnostic guidelines [5,6,8] were also reviewed with the following standardised assessment tools recommended: Movement ABC [6], BOTMP [6], Bruininks Oseretsky Test of Motor Proficiency Second Edition (BOT-2) [8], Alberta Infant Motor Scale (AIMS) [6], Peabody Developmental Motor Scales Second Edition (PDMS - 2) [6,8], Miller Function and Participation Scales (M –FUN) [8] and the Bayley Scales of Infant Development Second Edition (BSID II) [5]. Further review of these assessment tools found only the BOT-2 and Movement ABC were applicable based on age appropriateness, cultural suitability and comprehensive assessment design.

The phone survey of Australian Children’s Hospital Physiotherapy Outpatient Departments (n = 6) recommended the same two motor assessments in their revised versions – Movement ABC Second Edition (Movement ABC −2) [15] and the BOT-2 [16]. Papers describing the clinimetric properties of each of these tools were reviewed [17-20] and their appropriateness for use in a remote Aboriginal community was considered.

Discussions with clinicians from international FASD services at the 4th International Conference on FASD, Vancouver, March 2011 unanimously concluded that the BOT-2 was the motor assessment tool of choice because of its comprehensive assessment design and sensitivity to detect motor impairment [16].

BOT-2 testing involves game-like motor tasks which capture the child’s interest and are not verbally complex [21] and therefore suitable for children of non-English speaking background. The authors report that it can identify motor deficits in individuals with “mild to moderate” motor impairment and is validated and reliable for assessing subjects with “mild to moderate” mental retardation [16]. Importantly, both aspects fit the profile of children with a FASD diagnosis. The earlier version, the BOTMP [22], is a widely used standardised assessment tool with a long history of use in clinical practice and research. It is often used as the standard for the criterion validation of other motor tests [23]. Both CF and SF versions report score outcomes in percentile ranks thus satisfying requirements for use in internationally recognised FASD diagnostic processes. Furthermore, the motor activities incorporated within the BOT-2 include GM tasks that assess hopping, jumping, running, ball skills, balance, strength, and co-ordination and FM tasks that assess precision, integration and manual dexterity through drawing, writing, and functional tasks such as threading blocks. Through interviews with community members we established that these motor tasks are consistent with motor activities of Fitzroy Valley children at school and in recreational time. As yet, the reliability of the BOT-2 CF or BOT-2 SF has not been established either in children exposed to alcohol *in utero* or for the motor assessment of Australian Aboriginal children.

The BOT-2 authors report that BOT-2 SF was designed as a screening tool to identify children with motor deficits who may benefit from further comprehensive testing for diagnostic purposes or intervention activities [16]. Whilst the Lililwan Project FASD prevalence study used the more comprehensive BOT-2 CF, the reliability study used the shorter BOT-2 SF in order to minimise assessment fatigue as the reliability study was conducted in addition to the concurrent FASD prevalence study. Pilot testing had indicated that a reliability study involving the BOT-2 CF may be too exhausting given each child participating in the Lililwan Project underwent approximately 6 hours of interdisciplinary assessments over two days (including the BOT-2 CF assessment) as part of the FASD diagnostic process [24]. Even though the Lililwan Project occurred over a 6 month period, the assessment team had little flexibility in timetabling assessments, and this was compounded by the remoteness of most communities. The Lililwan Project team visited each community for a limited time, during which assessments, data entry, FASD diagnosis (and other diagnoses) and individual management plans needed to be completed. For these reasons a limited sample (n = 30) of the Lililwan Project (n = 108) was recruited for the reliability testing using the shorter BOT-2 SF as this measure takes approximately 20 minutes to complete compared with 60 minutes for the BOT-2 CF. The 14 test items in the BOT-2 SF are included within the BOT-2 CF, enabling comparison of these 14 key items between the BOT-2 SF and the BOT-2 CF to determine the test-retest reliability. Correlation between the BOT-2 CF and SF is not provided by the BOT-2 authors [16]. However, a study using the earlier BOTMP version reported a high correlation between the CF and SF total composite scores using Pearson’s product–moment coefficients [*r* = 0.85 (95% CI, 0.80 – 0.89)] [25].

### Measurement of change

Of further benefit is the provision of cut-offs which indicate true change in a subject’s performance at a second assessment point attributable to intervening factors, such as a therapy program, rather than measurement error. The standard error measure (SEM) reflects the degree to which a measurement can vary as a result of error in the measurement process [26]. The minimal detectable change (MDC) shows which changes fall outside the measurement error range ie changes greater than the MDC can be attributed to real change and not to measurement error [27]. The SEM and MDC are based on test-retest reliability in stable persons. They are both estimates of the extent of measurement error based on the standard deviation (SD) and reliability value, and are readily interpretable as they are given in the same units of measurement as the instrument under examination [26,27]. As the BOT-2 SF is a concise motor assessment designed as a screening tool, these estimates are calculated for the BOT-2 SF outcome scores rather than from the individual 14 subtest items.

The aims of this study were to:

1. determine the inter-rater and test-retest reliability of the BOT-2 SF amongst a convenience sample of children (n = 30) selected from the group of children born in 2002 or 2003 participating in the Lililwan Project cohort (n = 108) where over 50% of mothers drank alcohol during pregnancy.

2. estimate the SEM and MDC for the BOT-2 SF score sheet outcomes (standard scores and percentile ranks).

## Methods

### Setting

The study was conducted in the remote Fitzroy Valley of north Western Australia, which is located 2,500 km North of Perth, and 400 km East of Broome. This area has a population of approximately 4,500 people including the town of Fitzroy Crossing and the majority of the population is Aboriginal. There are approximately 45 remote communities within a 200 km radius of Fitzroy Crossing town representing the language groups of Bunuba, Walmajarri/Wangkatjungka, Gooniyandi and Nyikina peoples [28]. Kimberley Kriol is the most commonly spoken language but traditional Aboriginal languages (Bunuba, Walmajarri/Wangkatjungka, Gooniyandi and Nyikina) and Standard Australian English (SAE) are also used. School curriculums are taught in SAE.

### Context

The “Lililwan Project,” is a population based study which used an active case ascertainment approach for assessing FASD prevalence. The families of all children born in 2002 and 2003 in the Fitzroy Valley were contacted for consent to participate in this study as described in the Lililwan Project study protocol [24]. Historical information to assist with the diagnosis of FASD was obtained by interviewing parents and carers using a specifically developed questionnaire [29]. Over a six month period from May – November 2011, an interdisciplinary team conducted comprehensive health and development assessments in 108 children as part of the FASD diagnostic process for the Lililwan Project.

### Study design and participants

This study design was a prospective inter-rater and test-retest reliability study design. A convenience sample was selected for reliability testing of the BOT-2 from the overall Lililwan Project cohort (n = 108) where over 50% of mothers drank alcohol during pregnancy. Children were selected based on their availability to participate. All children (n = 30) were of Aboriginal and Torres Strait Island background and no child had a known disorder that would affect motor performance. Table 1 provides further information about the sample characteristics. All children were assessed blind to knowledge of a FASD diagnosis and PAE.

**Table 1:** Sample characteristics (n = 30)

**Sample characteristics**	**Outcome**
Boys	60% (n = 18)
Age at first test time point: mean SD (range)	8 yrs, 5 m (7 yrs, 6 m - 9 yrs, 6 m )
Age at second test time point: mean (range)	8 yrs, 7 m (7 yrs, 7 m - 9 yrs, 7 m )
Median retest time (range)	45.5 days (11 – 114 days)
Children unable to complete BOT-2 SF or CF assessment	0
Children who performed SF first	67% (n = 20)
RHS dominant drawing	87% (n = 26)

*SD* standard deviation, % percentage, *yrs* years, *m* months, *RHS* right hand side, *SF* Short Form, *CF* Complete Form.

### Measures

#### BOT-2 SF

The BOT-2 is a norm referenced standardised motor assessment available in a Complete Form with 53 items or a Short Form with 14 items selected from the Complete Form. It is suitable for use in children aged 4 to 21 years [16]. Both versions are categorised into four composite motor domains each containing two motor subtests i.e. **1. Fine Manual Control**: Fine Motor Precision, Fine Motor Integration; **2. Manual Coordination**: Manual Dexterity, Upper-Limb Coordination; **3. Body Coordination**: Bilateral Coordination, Balance; **4. Strength and Agility**: Running Speed and Agility, Strength. The “strength” subtest has two options for performing the “push-up” test and for our study the knee “push-up” option was chosen. Total motor composite and subtest measures are available as a raw score, standard score, percentile rank and descriptive category (“well below average”, “below average”, “average”, “above average and “well above average”). Gender specific norms were used for scoring as the BOT-2 authors report these as being more accurate than combined gender norms [16]. The BOT-2 SF was selected for the reliability study for reasons previously mentioned. It’s maximum Total Point Score or raw score is 88.

#### Measures of change

The following measures were calculated for the BOT-2 SF test-retest reliability outcome scores:

(i) ***SEM:*** The SEM was calculated using the following equation; SEM = SD * √(1 – r ) where SD is the pooled Standard Deviation; and r is the intraclass correlation co-efficient [27].

(ii) ***MDC:*** The MDC was calculated using the following equation; MDC_95_ = 1.96 * √2 * SEM where 1.96 is the z score associated with a 95% confidence interval and √2 reflects the variance of the two measures involved [27].

### Procedure

Reliability testing was always performed on a different day to the interdisciplinary assessments to prevent fatigue impacting on reliability test results. The BOT-2 SF was administered in standardised conditions according to the test manual and kit [16]. Three assessors (BL, GH and RD) undertook three hours of training prior to commencement of the study. They were experienced physiotherapists (BL and GH, with BL being a specialist paediatric physiotherapist) and an occupational therapist (RD) who had worked in paediatric populations, including with Aboriginal children in the Fitzroy Valley. Training consisted of watching the “BOT-2 Training Video” [30] and performing trial BOT-2 SF assessments on two children, followed by discussions amongst the assessors to resolve any differences to improve reliability. Assessors were blinded to the child’s PAE at the time of GM assessment and scoring. BOT-2 test instructions to children were taught through verbal instruction and demonstration. If the child did not understand test instructions then the test item was demonstrated by the assessor. Community navigators (local Aboriginal community members) who spoke Kimberley Kriol, local Aboriginal languages and SAE were present throughout the assessment. Their main role was to assist with communication to ensure that test results reflected best motor performance and were not invalidated or diminished by language or cultural barriers. Assessments were conducted in a formal but playful manner to maximise participation and attain the child’s best motor performance.

To determine ***inter-rater reliability,*** each subject was assessed using the BOT-2 SF and rated by both assessors (BL and GH) simultaneously. Assessors alternated in providing test instructions to the subject with both assessors simultaneously completing separate copies of the BOT-2 SF score sheet. Assessors were blinded to each other’s results and no consultation between them was permitted. Data were entered independently by each assessor into the “BOT-2 Assist Scoring and Reporting System” software (2007, Pearson Assessments) to determine BOT-2 scores and later entered by a blinded independent research assistant into the Lililwan Project database.

***Test-retest reliability*** was determined by re-testing the subject using the BOT-2 CF during the later Lililwan Project diagnostic interdisciplinary assessments. From this test the 14 relevant items were extracted for comparison with the same items from the original BOT-2 SF test. The BOT-2 CF assessment was conducted within four months before or after the BOT-2 SF reliability assessment. The GM components (Upper Limb Coordination, Bilateral Coordination, Balance, Running Speed and Agility, and Strength) were tested by a physiotherapist (BL) and the fine motor components (Fine Motor Precision, Fine Motor Integration and Manual Dexterity) were tested by an occupational therapist (RD).

Assessments were conducted outdoors in shaded conditions within the grounds of community schools. Wherever possible, assessments were completed in the morning to assist children with concentration and to avoid the high midday temperatures common in the area which sometimes exceed 35 degrees Celsius. Consideration was given to the school timetable, taking care to avoid periods such as recess, lunch and school assemblies to minimise distractions from other children during BOT-2 assessments.

### Statistical analysis

The BOT-2 scores are considered continuous data with equal intervals between data points on the scale. Statistical analysis of BOT-2 SF, to obtain inter-rater and test-retest reliability, was performed for the 14 subtest items and key BOT-2 score sheet outcome items (total point score, standard score, percentile ranks). Inter-rater and test-retest reliability was calculated between assessors for these items using the intraclass correlation coefficient ICC(2,1) as the main measure. The ICC measure, however, is not robust as it can be severely affected by outliers that produce large discrepancies or disagreements at extreme points of range, or poor variance amongst the test item scores [31]. Two other measures of agreement were calculated to compensate for this: the percentage exact agreement (PEA) and percentage close agreement (PCA). PEA is the proportion of subjects in which both assessors agree exactly on the score for a test item and is a more precise measure of agreement than PCA. PCA is similar but includes the subjects where assessors differed by a single point ie ± 1 [31]. They complement the ICC agreement measure as they purely count agreements and disagreements, irrespective of the size, and are particularly useful when outliers are present amongst the data set. They may overestimate true reliability as they don’t discount the proportion of agreement that is potentially due to chance alone, hence the ICC is the preferred estimate of agreement [32]. If ICC values are low and PEA and PCA are high then it is likely that outliers exist or that the range of scores or score variance is limited within the data set [31]. Poor reliability is present when the ICC values as well as the PEA and PCA are low. PEA was determined by dividing the number of exact agreements by the total number of paired judgements, expressed as a percentage. PCA was determined by dividing the number of close agreements (defined as where differences between the paired judgments ranged from – 1, including 0, to + 1) by the total number of paired agreements, expressed as a percentage.

Interpretation of the ICC statistic strength of agreement was based on the method proposed by Fleiss for continuous data: an ICC value above 0.75 indicates *excellent reliability*, 0.4 –0.75 indicates *fair to good reliability* and values below 0.4 indicate *poor reliability*[33]. All statistical analyses were performed using IBM SPSS Statistics Standard Grad Pack Shrinkwrap version 21.0 (IBM Corporation). Statistical significance was set at p ≤ 0.05. The sample size of 40 participants was determined to provide sufficient power to find a correlation coefficient of 0.7, with confidence intervals ranging from 0.5 to 0.9.

### Ethics approval

Ethics approval was granted for all stages of this study by the University of Sydney Human Research Ethics Committee (approval numbers 12527, 13187, 13551), the Western Australian Aboriginal Health Information and Ethics Committee (approval numbers 271-01/10, 319-10/10, 344-04/11), the Western Australian Country Health Service Board Research Ethics Committee (approval numbers 2010:01, 2010:28, 2011:04) and the Kimberley Aboriginal Health Planning Forum Research Sub-committee (approval numbers 2010–001, 2010–001, 2010–001).

## Results

Thirty Aboriginal children (18 male, 12 female: mean age 8.8 years) were assessed at eight remote Fitzroy Valley communities between May to October, 2011. Table 1 shows the characteristics of the participants and the testing schedule. FASD diagnostic outcomes were equally distributed in the reliability sample comparative to the cohort sample.

### Inter-rater reliability

Reliability was calculated for two assessors simultaneously assessing each of the 30 participants and the results are displayed in Table 2. The ICC(2,1) and PEA for test items ranged between 0.34 to 1.00, and 100% to 43% respectively. Subtest items with the highest ICC(2,1) and PEA were *“transferring pennies*” ICC(2,1) 1.00 (95% Confidence Interval (CI), 1.00 – 1.00), PEA 100% and “*dropping and catching a ball – both hands*” ICC(2,1) 1.00 (95% CI, 1.00 – 1.00), PEA 100%. Subtest items with the lowest ICC(2,1) and PEA were *“one-legged stationary hop”* ICC(2,1) 0.49 (95% CI 0.16 – 0.72), PEA 43% and *“jumping in place - same sides synchronised”* ICC(2,1) 0.34 (95% CI -0.03 – 0.62), PEA 83%*.* Importantly the PCA ranged from 83% to 100% across all 14 subtest items, indicating agreement within one point amongst assessors between 83% and 100% of all scores. Table 3 shows that 58% of the ICC’s subtest items demonstrated *excellent* reliability and 79% *fair to good* reliability or higher.

**Table 2:** Inter-rater and test-retest reliability for BOT-2 SF subtest and score sheet outcome items (n = 30)

**Subtest**	**Subtest item *(NB: numbering of items relates to CF)***	**Percentage exact agreement%**		**Percentage close agreement%**		**ICC (2,1)**		**95% CI**	
		**Inter-rater**	**Test-retest**	**Inter-rater**	**Test-retest**	**Inter-rater**	**Test-retest**	**Inter-rater**	**Test-retest**
**1. Fine Motor Precision**	3. Drawing lines through paths- crooked	97	60	100	80	0.66	0.13	0.40 - 0.82	- 0.13 - 0.42
	6. Folding paper	83	57	97	87	0.92	0.76	0.84 - 0.96	0.55 - 0.88
**2. Fine Motor Integration**	2. Copying a square	80	57	100	97	0.89	0.00	0.79 - 0.95	- 0.36 - 0.36
	7. Copying a star	50	33	90	70	0.80	0.25	0.61 - 0.90	- 0.13 - 0.56
**3. Manual Dexterity**	2. Transferring pennies	100	37	100	93	1.00	0.48	1.00 - 1.00	0.16 - 0.71
**4. Bilateral Co-ordination**	3. Jumping in place - same sides synchronized	83	70	90	90	0.34	- 0.066	- 0.03 - 0.62	- 0.41- 0.29
	6. Tapping feet and fingers- same sides synchronized	97	90	100	93	N/A*	- 0.032	N/A*	- 0.38 - 0.33
**5. Balance**	2. Walking forward on a line	97	97	100	100	N/A*	N/A*	N/A*	N/A*
	7. Standing on one leg on a balance beam - eyes open	87	57	97	83	0.54	0.17	0.23 - 0.75	- 0.15 - 0.47
**6. Running Speed and Agility**	**3**. One-legged stationary hop	43	27	83	77	0.49	0.25	0.16 - 0.72	- 0.091 - 0.55
**7. Upper-limb Co-ordination**	1. Dropping and catching a ball - both hands	100	87	100	97	1.00	- 0.041	1.00 - 1.00	- 0.39 - 0.32
	6. Dribbling a ball - alternating hands	83	73	93	83	0.85	0.023	0.72 -0.93	- 0.34 - 0.38
**8. Strength**	2a. Knee push ups	60	33	100	77	0.87	0.31	0.74 - 0.93	- 0.065 - 0.60
	3. Sit ups	77	27	90	67	0.86	0.26	0.73 -0.93	- 0.11 - 0.57
**BOT-2 Score Sheet Outcomes**		**Percentage exact agreement%**		**Percentage close agreement%**		**ICC (2,1)**		**95% CI**	
1. Total point score*(Raw score)*						0.92	0.62	0.84 -0.96	0.34 – 0.80
2. Standard score*(Standardised score for gender* &*age)*						0.89	0.73	0.78 - 0.95	0.50 - 0.86
3. Percentile rank*(%)*						0.88	0.71	0.77 - 0.94	0.48 - 0.85

*ICC* intraclass correlation coefficient.

*CF* comprehensive form.

* ICC unable to be computed because there was poor variance.

**Table 3:** **Interpretation of inter-rater reliability for BOT-2 SF subtest and score sheet outcome items results based on Fleiss’s method of interpretation **[33]**for ICC (2,1) (n = 30)**

**Fleiss method of interpretation for ICC (2,1)**	**Subtest items**	**No. of subtests (%)**
Excellent reliability **(≥0.75)**	• Folding paper	8 (58%)
	• Copying a square	
	• Copying a star	
	• Transferring pennies	
	• Dropping and catching a ball – both hands	
	• Dribbling a ball – alternating hands	
	• Knee push-ups	
	• Sit ups	
Fair to good reliability **(> 0.40 - < 0.75)**	• Drawing lines through Paths – crooked	3 (21%)
	• Standing on one leg on a balance beam – eyes open	
	• One leg stationary hop	
Poor reliability **(< 0.40 )**	• Walking forward on a line	3 (21%)
	• Jumping in place same sides synchronised	
	• Tapping feet and fingers same sides synchronised	
	**Score sheet outcome items**	**No. of outcomes (%)**
Excellent reliability **(≥ 0.75)**	• Total point score	100% (3/3)
	• Standard score	
	• Percentile rank	

*ICC* intraclass correlation coefficient.

The ICC’s for the BOT-2 Score Sheet Outcomes which summarise the 14 subtests ranged from 0.88 to 0.92. They provide the key results of the BOT-2, showing the child’s rank against population norms. The percentile ranks may be compared against predefined cut-offs to assist in assigning a diagnosis of FASD. Table 3 shows that 100% of the outcome scores represented *excellent* reliability.

### Test-retest reliability

Test-retest reliability was calculated for the assessment of each of the 30 participants between two time points by predominantly the same assessor. The results are displayed in Table 2. The median time from test to retest period between tests was 45.5 days (range 11 – 114 days), this longer test -retest interval reflecting the logistics of conducting a study in remote communities. Subjects were assessed with the BOT-2 SF on one occasion and the BOT-2 CF on another occasion extracting the 14 BOT-2 SF items from the BOT-2 CF (67% of subjects were assessed with the BOT-2 SF at the first occasion). The ICC(2,1) and PEA for test items ranged between −0.07 to 0.76, and 27% to 97% respectively. Subtest items with the highest ICC(2,1) and PEA were “*folding paper*” ICC(2,1) 0.76 (95% CI, 0.55 - 0.88), PEA 57% and *“transferring pennies*” ICC(2,1) 0.48 (95% CI, 0.16 – 0.71), PEA 37%. Subtest items with the lowest ICC(2,1) and PEA were “*jumping in place – same sides synchronised*” ICC(2,1) -0.066 (95% CI, -0.41 – 0.29), PEA 70% and *“sit ups*” ICC(2,1) 0.26 (95% CI, -0.11 – 0.57), PEA 27%. Table 2 shows that the PCA ranged from 67% to 100% across all 14 subtest items despite 86% of the ICC(2,1) subtest scores showing *poor* reliability (Table 4). The ICC’s for the BOT-2 Score Sheet Outcomes which summarise the 14 subtests ranged from 0.62 to 0.73. Table 4 shows that 100% of the ICC(2,1) outcome scores indicated *fair to good* reliability.

**Table 4:** **Interpretation of test-retest reliability for BOT-2 SF subtest and score sheet outcome items results based on based on Fleiss’s method of interpretation **[33]**for for ICC (2,1) (n = 30)**

**Fleiss’s method of interpretation for ICC (2,1)**	**Subset categories**	**No. of subtests (%)**
Excellent reliability	• Folding paper	7% (1/14)
**(≥ 0.75)**		
Fair to good reliability	• Transferring pennies	7% (1/14)
**(> 0.40 - < 0.75)**		
Poor reliability	• Drawing lines through paths – crooked	86% (12/14)
**(< 0.40 )**	• Copying a square	
	• Copying a star	
	• Jumping in place same sides synchronised	
	• Tapping feet and fingers same sides synchronised	
	• Walking forward on a line	
	• Standing on one leg on a balance beam – eyes open	
	• One legged stationary hop	
	• Dropping and catching a ball – both hands	
	• Dribbling a ball – alternating hands	
	• Knee push-ups	
	• Sit ups	
	**Score sheet outcome items**	**No. of outcomes (%)**
Fair to good reliability	• Total point score	100% (3/3)
**(> 0.40 - < 0.75)**	• Standard score	
	• Percentile rank	

*ICC* intraclass correlation coefficient.

**Figure 1:**
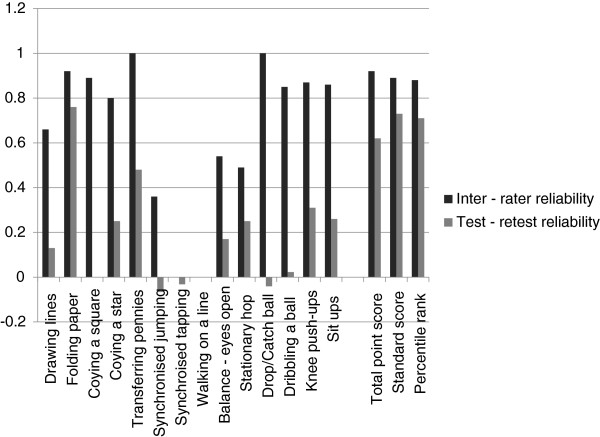
Comparison of inter-rater and test-retest reliability ICC (2,1) results.

Figure 1 compares the ICC(2,1) values between the inter-rater and test-retest reliability, showing the stronger agreement for inter-rater reliability.

### Measures of change

The SEM and MDC for the Total Point Score (raw score) was 2.21 and 6.12. The SEM and MDC for the Standardised Score (adjusted for gender and age) was 2.06 and 5.71. The SEM and MDC for the Percentile Ranks was 7.61 and 21.09.

Figure 1 compares the ICC(2,1) values between the inter-rater and test-retest reliability, showing the stronger agreement for inter-rater reliability.

## Discussion and conclusions

This is the first study to evaluate the reliability of the BOT-2 SF in Aboriginal children living in remote Australian communities, where many children have been exposed prenatally to high levels of maternal alcohol consumption. The results of this study suggest that the BOT-2 SF is a reliable standardised assessment tool for use in the context of assessing motor proficiency in Australian Aboriginal children, including children with PAE, as acceptable inter-rater and test-retest reliability was established. Importantly, all subjects were able to complete both the BOT-2 SF and the longer BOT-2 CF assessments, including subjects with a FASD diagnosis.

Factors which affected the results of this reliability study included the length of the retest period, the outliers and poor score variance. Reasons for the lower test-retest reliability scores compared to the higher inter-rater reliability measures are mostly due to the long intervening retest period. Inter-rater reliability scores reflect the child’s performance at one point in time as judged by two raters, whereas the test-retest measures are based on the child’s performance on two occasions, separated by a median of 45.5 days. Data analysis revealed that ICC values were more affected by constrained score variance than outliers in both inter-rater and test-retest reliability. Inspection of the data revealed only a small number of outliers, all within the test-retest reliability data, and mainly reflecting some improvement in the child’s performance on the second test occasion. In instances when outliers or poor score variance was present, the estimates of agreement for the affected subtest items were better indicated by PEA and PCA scores than ICC’s values. For example, in the test-retest reliability, the low ICC’s for many subtests are complimented by high PCA’s indicating that either a large outlier or poor variation in the score results may be contributing to the low ICC values. Similarly, in the inter-rater reliability there were two items (“walking forward on a line” and “tapping feet and fingers same sides synchronised”) where ICC values could not be calculated because the motor task was too easy and most subjects achieved a maximum score. This resulted in extremely low score variance but high PCA and PEA values. It is hypothesised that these very high subtest item scores may reflect either the construct design of the BOT-2 or the highly developed motor abilities of Aboriginal children arising from the physical activities they engaged in during recreational time and integral to Aboriginal culture. As yet there are no existent normative data describing the motor skills of Australian Aboriginal children or studies using standardised assessment tools such as the BOT-2 CF or SF for comparison with our data. Another study reported ceiling effects in items from the BOT-2 SF in a cohort of 6 – 10 year olds (n = 113) from a USA Midwestern town including “walking forward on a line” and “drawing lines through paths – crooked” [34]. This suggests a problem with construct design. Other ceiling effects may have a cultural basis requiring clarification through further research.

The inter-rater reliability scores are of particular interest as they are not biased by maturation of the child. If we consider the least reliable subtest items in these scores they are those that have low ICCs, low PEAs and low PCAs, where the rater may have had difficulty assigning a score. The activities were highly dynamic, fast activities; “one-legged stationary hop” and “jumping in place - same sides synchronised”. It is recommended that these items have a particular focus during BOT-2 SF training to strengthen standardisation of scoring.

This study has several strengths. A key strength was that the BOT-2 SF was conducted over eight different remote assessment locations, verifying that it is feasible for use in other remote Australian Aboriginal communities. Significantly, the BOT-2 SF was found to be reliable within the population of interest, i.e. children with PAE and within an age group where motor impairment is likely to be encountered. Importantly, the children enjoyed completing the BOT-2 SF tasks and all children finished all components of the assessment. Furthermore, the BOT-2 SF is a well-known assessment tool used by physiotherapists and occupational therapists within Australia and internationally. With reliability now established this standardised assessment may be useful for documenting deficits in GM and FM function when evaluating children with PAE or motor proficiency amongst Australian Aboriginal children in remote communities.

There are three possible limitations to the methodology of this study, the first of which may have contributed to the lower test-retest reliability results for equivalent items shown in Figure 1. There was a median period of 45.5 days (range 11 – 114 days) between the test-retest assessments with the majority of children retested after 8 weeks (n = 12), followed by 2-4 weeks (n = 10), 4-8 weeks (n = 7) and within a fortnight (n = 1). Consequently children had aged by the time of the second test and motor performance may have improved during this time. Factors likely to cause variability of performance and ‘target drift’ over this period include behavioural and motivational differences, motor improvements and health influences. The resultant outcome scores denoting *fair to good* test-retest reliability indicate the robust construct of the BOT-2 SF despite these limitations. In ideal study conditions the test-retest time interval would be less [32] however the study design was realistic to the logistics of the remote conditions and the results are considered a cautious estimate for the BOT-2 SF test–retest reliability. A second limitation of this study was that the preferred, more comprehensive BOT-2 CF was not used at both time points to assess subjects. Pilot testing indicated that children may have difficulty completing the more extensive CF a second time in the context of 6–8 hours of interdisciplinary assessments. Therefore it was decided to use the SF and compare this to the relevant items in the CF performed as part of the Lililwan Project. A third limitation of our study was the difficulty involved in recruiting sufficient subjects for the reliability study from remote communities. Our original power calculations had suggested that 40 subjects would be optimal, however it was only possible to recruit 30 children to the study.

There are two published studies which report on the reliability of the BOT-2. In one, the BOT-2 manual authors report the BOT-2 CF and SF are reliable measures of motor skill ability when evaluating internal consistency and test-retest reliabilities in healthy subjects aged 4–21 years [16]. A second reliability study for the BOT-2 CF demonstrated excellent internal consistency and test-retest reliability in the measurement of motor proficiency in children and adolescents with intellectual disability (ID) aged from 4 to 12 years [35]. Apart from information provided in the BOT-2 Manual [16], the study described in this paper is the only one assessing reliability of the BOT-2 SF and the only study focused on children known to be exposed *in-utero* to alcohol. It shows that the BOT-2 SF has strong inter-rater and moderate test-retest reliability in the assessment of Australian Aboriginal children aged 7 to 9 years living in remote locations including those with PAE and elements of ID and/or attention problems. MDC values have not been reported elsewhere for the BOT-2 SF for comparison. The precision of MDC and SEM values is likely to improve with a shorter retest period. The percentile ranks of the score sheet outcomes are very sensitive to changes in the standardised score; hence the large MDC of 21.12 is expected despite the smaller SEM of 7.06. The raw score (SEM 2.21, MDC 6.12) and the standardised score (SEM 2.06, MDC 5.71) are proposed as more meaningful measures to detect true change in motor proficiency with the MDC’s and SEM being more closely correlated. The raw score MDC equates to a 7% change in the maximum total point score.

Future research should include assessment of the reliability of the BOT-2 CF in populations of children exposed prenatally to alcohol and across a wider age group. BOT-2 SF studies investigating test-retest reliability should be performed using a short time interval to avoid potential change in results due to maturation. In comparison with the BOT-2 SF which was designed as a screening tool, the BOT-2 CF is a more comprehensive - motor assessment tool and therefore is recommended for use in FASD diagnostic processes where children might have a wide range of capabilities. Assessments ideally should be performed by experienced paediatric physiotherapists and occupational therapists [36]. Knowledge developed in this study will be transferable to other Australian Aboriginal communities seeking to improve diagnostic capacity for FASD. This study demonstrates that the BOT-2 SF is a reliable assessment tool for use with Aboriginal children living in remote communities in the evaluation of motor impairment and in those exposed *in utero* to alcohol.

## Abbreviations

BOT-2: Bruininks-Oseretsky Test of Motor Proficiency Second Edition; CF: Complete form; CI: Confidence interval; CNS: Central nervous system; FASD: Fetal alcohol spectrum disorder; FM: Fine motor; GM: Gross motor; ICC: Intraclass correlation coefficient; ID: Intellectual disability; MDC: Minimal detectable change; Movement ABC-2: Movement assessment battery for children second edition; PAE: Prenatal alcohol exposure; PCA: Percentage close agreement; PEA: Percentage exact agreement; SAE: Standard Australian English; SD: Standard deviations; SEM: Standard error measure; SF: Short form.

## Competing interests

The authors declare they have no competing interests.

## Authors’ contributions

JL, MLF and BRL conceived and designed the study. JL, EJE, JPF, MLF, MC and JO obtained ethics approval for the study. BRL conducted the literature review and contacted national and international paediatric and FASD networks to identify the BOT-2 assessment tool. MH and BRL consulted with participant communities. BRL and GH conducted participant recruitment and data collection for BOT-2 SF. BRL and RD collected data for the BOT-2 CF. RA, BRL and MLF analysed the data and BRL, RA, JL, EJE, and MLF contributed towards the interpretation of results. BRL wrote the initial drafts of the manuscript. All authors read, edited and approved the final manuscript.

## Authors’ information

MC, JO and MH are Aboriginal leaders from the Fitzroy Valley communities. JL , EJE, JPF, JO and MC are Chief Investigators for the Lililwan Project. BRL and JPF are PhD candidates with The University of Sydney, New South Wales. MLF is a Senior Research Fellow for the George Institute for Global Health. RA is a Senior Lecturer in the Faculty of Health Sciences, University of Sydney. RD is a PhD candidate with the School of Public Health, Curtin University of Technology, Perth, Western Australia. JO and MH are Master’s candidates with the University of Notre Dame, Broome, Western Australia. GH is a clinical physiotherapist experienced in working in remote Australian Aboriginal communities.

## Pre-publication history

The pre-publication history for this paper can be accessed here:

http://www.biomedcentral.com/1471-2431/13/135/prepub
